# Adaptive Sensor Fusion for Robust Perception in Dense Fog: A Gated Vision and LiDAR Integration Framework

**DOI:** 10.3390/s26123728

**Published:** 2026-06-11

**Authors:** Fengyuan Zhang, Zixuan Guo, Jianbo Ding, Jingyun Yang, Wenhe Liu

**Affiliations:** 1 Tandon School of Engineering, New York University, New York, NY 10010, USA; fz2240@nyu.edu; 2 Steinhardt School, New York University, New York, NY 10010, USA; zg2759@nyu.edu; 3 SonicWall, Milpitas, CA 95035, USA; jianboding@ieee.org; 4 David A. Tepper School of Business, Carnegie Mellon University, Pittsburgh, PA 15213, USA; claudey@alumni.cmu.edu

**Keywords:** gated imaging, LiDAR, adaptive sensor fusion, uncertainty estimation, 3D object detection, adverse weather, dense fog, autonomous driving

## Abstract

Autonomous driving systems face critical perception failures in dense fog, where conventional RGB cameras suffer from severe degradation due to atmospheric scattering and reduced visibility. This paper presents an adaptive multi-modal fusion framework that synergistically integrates gated imaging with 3D LiDAR point clouds to achieve robust obstacle detection under visibility conditions as low as 50 m. Unlike standard cameras that passively capture scattered ambient light, gated cameras employ time-synchronized active illumination to physically filter backscattered photons, preserving structural features even in low-visibility scenarios. We propose a novel Adaptive Feature-Weighting Network (AFW-Net) that dynamically adjusts sensor modality contributions based on real-time environmental degradation assessment. The framework incorporates three key innovations: (1) a cross-modal feature extraction module that exploits the complementary physical properties of gated imaging and LiDAR, (2) an attention-based adaptive fusion mechanism that quantifies per-modality reliability through uncertainty estimation, and (3) a degradation-aware training strategy using weather-specific augmentation. Extensive experiments on the Princeton Automated Driving Dataset demonstrate that our approach maintains detection average precision (AP) above 82% under dense fog conditions (50 m visibility), representing a 23.7% improvement over state-of-the-art RGB-LiDAR fusion methods that exhibit substantial performance degradation to 58.4% AP. Ablation studies validate the necessity of each component, and cross-dataset evaluation confirms the generalization capability of the proposed framework. The adaptive weighting mechanism proves particularly effective, dynamically rebalancing modality contributions across the gated imaging and LiDAR branches while maintaining LiDAR geometric constraints. This work establishes a robust perception paradigm for safety-critical autonomous systems operating in low-visibility environmental conditions.

## 1. Introduction

The rapid advancement of autonomous driving systems has positioned perception as the cornerstone of safe and reliable vehicle operation. Modern autonomous vehicles rely heavily on multi-modal sensor suites, typically comprising RGB cameras, LiDAR, and radar, to construct comprehensive environmental representations [1]. However, the performance of these perception systems degrades significantly under adverse weather conditions such as dense fog, heavy rain, and snow, where atmospheric scattering and particle occlusion severely compromise sensor reliability [2,3]. Studies have shown that conventional RGB cameras suffer from exponential visibility degradation in fog with meteorological optical ranges below 100 m [3], while LiDAR sensors experience substantial point cloud sparsification and noise amplification due to raindrop reflections and snowflake interference [4,5].

The safety-critical nature of autonomous driving mandates robust perception capabilities across all operational conditions. According to transportation safety statistics, adverse weather conditions contribute to approximately 21% of vehicle crashes and 16% of crash fatalities annually in the United States alone [6]. Current state-of-the-art perception systems, predominantly based on RGB-LiDAR fusion architectures [7,8], exhibit pronounced performance degradation when visibility drops below 50 m—a common occurrence in dense fog or heavy precipitation scenarios. This phenomenon, often characterized by a cliff-edge performance drop rather than graceful degradation, poses unacceptable risks for safety-critical applications.

Recent research has explored various strategies to enhance perception robustness in adverse weather. Domain adaptation techniques attempt to bridge the distribution gap between clear and degraded conditions through synthetic data augmentation [3] or adversarial training [9]. Statistical filtering methods have been developed to remove weather-induced artifacts from LiDAR point clouds [10]. However, these approaches face a shared limitation: they attempt to recover information that has been physically lost due to atmospheric scattering and absorption. When photons are scattered before reaching the sensor, no amount of computational post-processing can fully reconstruct the original scene structure.

Gated imaging technology addresses these physical limitations of passive sensing from a different angle. Unlike conventional cameras that continuously integrate all incoming photons, gated cameras employ time-synchronized active illumination paired with nanosecond-precision electronic shuttering [2,11]. This temporal gating mechanism enables selective capture of photons that have traveled the direct path from illumination source to target and back, while rejecting the majority of scattered photons from atmospheric particles. The physical principle underlying this capability is as follows: direct-path photons arrive earlier than multiply scattered photons due to their shorter optical path length. By opening the camera shutter only during a narrow temporal window corresponding to a specific distance range, gated imaging suppresses backscatter at the hardware level, preserving scene structure even in dense fog with visibility as low as 30–50 m.

Despite the promising physical properties of gated imaging, several challenges impede its direct adoption in autonomous driving systems. First, gated cameras require active illumination, which introduces range limitations and potential interference in multi-vehicle scenarios. Second, the temporal gating process captures only a depth slice of the scene per frame, necessitating multiple acquisitions or sophisticated reconstruction algorithms for complete scene coverage [11]. Third, and most critically, no single sensor modality provides optimal performance across all weather conditions—gated imaging excels in fog but offers no inherent advantage in clear weather, while LiDAR maintains geometric accuracy in many conditions but struggles with precipitation [12,13].

The key premise of this work is that robust all-weather perception requires adaptive fusion that dynamically adjusts sensor contributions based on real-time environmental conditions. Rather than treating different modalities as equally reliable throughout all scenarios, a fusion model should allocate greater weight to the sensors whose features are most reliable given the current degradation state. This principle motivates our proposed Adaptive Feature-Weighting Network (AFW-Net), which learns to assess per-modality reliability through uncertainty estimation and adjusts fusion weights accordingly.

We position our contribution within the body of confidence-aware and uncertainty-aware sensor-fusion research in autonomous driving. Prior fusion methods can be broadly grouped into three categories. Fixed-weight schemes [2] combine modalities with predetermined, condition-independent coefficients. Attention- and transformer-based schemes [14,15] learn data-dependent cross-modal interactions but optimize for average-condition accuracy and do not explicitly model the reliability of each modality under degradation. A third line of work estimates predictive uncertainty—typically at the output (bounding box) level—to calibrate detection confidence. Our framework differs from all three in two respects. First, we estimate uncertainty at the feature level for each modality and use it directly as the signal that drives fusion-weight allocation, rather than only calibrating final detections. Second, the uncertainty estimator is supervised by a teacher–student distillation objective (Section 4.4) so that the estimated uncertainty reflects deviation from clean-condition feature targets, giving it a concrete operational meaning rather than being a free latent variable. To our knowledge, no prior gated-LiDAR fusion work couples feature-level uncertainty estimation with adaptive weight allocation in this way.

Compared to existing multi-modal fusion methods that either use fixed fusion weights [2] or assume uniform sensor reliability across conditions [14,15], our work makes three distinct departures from this prior literature. First, we introduce a learned, uncertainty-driven adaptive weighting mechanism that dynamically rebalances sensor contributions at the feature level; to our knowledge, no prior gated-LiDAR fusion work provides this capability. Second, we design a degradation-aware training strategy that implicitly teaches the network to recognize weather-induced feature corruption through synthetic augmentation, eliminating the need for explicit weather condition labels at inference time. Third, we provide a systematic empirical study indicating that adaptive gated-LiDAR fusion maintains robust performance under dense fog conditions where RGB-LiDAR methods suffer severe failure, achieving over 23% improvement in detection precision on the evaluated dataset.

Our approach addresses three research questions: (1) How can we effectively extract and fuse complementary features from gated images and LiDAR point clouds given their disparate data structures and physical sensing principles? (2) How can the system autonomously assess the reliability of each sensor modality in real-time without explicit weather condition labels? (3) How can we ensure that the fusion mechanism gracefully transitions between modality-dominant regimes rather than exhibiting discontinuous behavior?

To answer these questions, we make the following contributions:

- We propose a cross-modal feature extraction architecture that leverages specialized encoders for gated imaging and LiDAR while establishing geometric correspondence through camera-LiDAR projection. The gated image encoder employs a residual architecture with channel attention to capture both intensity and range information encoded in gated acquisitions, while the LiDAR encoder utilizes PointNet++ [16] with semantic abstraction to extract hierarchical geometric features.
- We introduce an attention-based adaptive fusion mechanism that computes per-modality confidence weights through learned uncertainty estimation. Unlike fixed-weight fusion schemes, this mechanism adjusts the contribution of each sensor based on feature-level uncertainty metrics, shifting reliance toward more reliable modalities as environmental conditions degrade.
- We design a degradation-aware training strategy that employs weather-specific data augmentation to simulate progressive visibility reduction in both gated and conventional imaging modalities. This strategy enables the network to learn the correlation between feature degradation patterns and environmental conditions without requiring explicit weather annotations during inference.
- We conduct experiments on the Princeton Automated Driving Dataset, which contains synchronized gated imaging, LiDAR, and GPS data captured under diverse weather conditions. On this dataset, AFW-Net maintains average precision above 82% under dense fog conditions (50 m visibility), outperforming RGB-LiDAR fusion baselines that degrade to 58.4% AP under the same conditions.

The remainder of this paper is organized as follows. Section 2 reviews related work in multi-modal fusion for autonomous driving, adverse weather perception, and gated imaging technologies. Section 3 provides necessary background on gated imaging principles and problem formulation. Section 4 details the proposed methodology, including network architecture, adaptive fusion mechanism, and training strategy. Section 5 presents the experimental results, ablation studies, and comparative analysis. Finally, Section 6 concludes the paper with discussion of limitations and future research directions.

## 2. Related Works

This section reviews prior research across three interconnected domains: multi-modal sensor fusion for 3D object detection, perception systems under adverse weather conditions, and gated imaging technologies for autonomous driving applications.

### 2.1. Multi-Modal Fusion for 3D Object Detection

Multi-modal fusion has emerged as a dominant paradigm in autonomous driving perception, leveraging complementary strengths of different sensor modalities to achieve robust 3D object detection. Early fusion approaches directly concatenate features from different modalities at the input level [17], while late fusion methods combine detection results from independent single-modality networks [18]. However, these naive fusion strategies fail to capture cross-modal correlations and often suffer from suboptimal performance when modality reliability varies.

Recent advances have focused on intermediate fusion schemes that enable deep feature-level interaction. MV3D [17] pioneered the use of multi-view representations by projecting LiDAR point clouds onto bird’s-eye view and front-view planes, fusing them with RGB features through a region-based network. PointPainting [7] introduced sequential fusion by augmenting each LiDAR point with semantic class scores from an image segmentation network, enabling the 3D detector to leverage rich semantic information from RGB images. MVX-Net [19] proposed a voxel-based fusion framework with PointNet [20] encoders for point cloud processing and convolutional encoders for image features.

More sophisticated fusion architectures exploit attention mechanisms to model cross-modal dependencies. SECOND [21] introduced sparse convolutional networks for efficient 3D detection from point clouds, while PointPillars [22] simplified the representation by encoding point clouds into vertical pillars. Building upon these foundations, CLOCs [23] and 3D-CVF [24] developed cross-modal alignment modules that establish dense correspondences between image pixels and 3D points. PointAugmenting [25] proposed a decorrelated feature augmentation strategy to enhance cross-modal feature learning.

Transformer-based architectures have recently gained traction for multi-modal fusion. TransFusion [14] employs cross-attention mechanisms to fuse image and LiDAR features in a unified framework. BEVFormer [15] constructs bird’s-eye view representations from multi-camera images using spatial and temporal transformers, representing a modern trend toward unified BEV-based perception architectures rather than traditional multi-modal fusion. However, these methods predominantly assume consistent sensor reliability across operating conditions, making them vulnerable to performance degradation when individual modalities fail under adverse weather.

A related line of research explicitly models predictive uncertainty to improve fusion robustness. Confidence-aware and probabilistic fusion methods estimate per-detection or per-region uncertainty and use it to weight or gate sensor contributions, drawing on aleatoric and epistemic uncertainty formulations from Bayesian deep learning. Most of these approaches, however, estimate uncertainty at the detection-output level and target average-condition calibration. In contrast, our method estimates uncertainty at the feature level for each modality and uses it directly to drive the fusion weights, with the estimator supervised against clean-condition feature targets so that the uncertainty has a concrete operational meaning under degradation (Section 4.3 and Section 4.4). This distinction is what allows the fusion weights to shift systematically as a modality degrades, rather than only recalibrating the final confidence scores.

### 2.2. Perception Under Adverse Weather Conditions

The degradation of perception systems in adverse weather has been extensively studied from both data-centric and algorithm-centric perspectives. Sakaridis et al. [3] pioneered the use of synthetic foggy datasets for semantic segmentation, employing atmospheric scattering models to generate training data with controlled fog density. ACDC [26] extended this work by collecting real-world data across fog, rain, snow, and nighttime conditions, establishing comprehensive benchmarks for adverse weather robustness.

For LiDAR-based perception, several studies have characterized weather-induced degradation patterns. Rasshofer et al. [13] conducted empirical analysis of automotive laser radar performance in rain, fog, and snow, quantifying the relationship between particle density and ranging error. Kutila et al. [4] performed controlled experiments demonstrating that heavy rain can reduce LiDAR effective range by up to 50%, while Filgueira et al. [12] analyzed point cloud density degradation as a function of weather severity. These observations motivated the development of snow and rain removal algorithms. Kurup and Bos [10] proposed DSOR, a statistical filter that identifies and removes snowflake-induced noise points based on spatial–temporal consistency. Hahner et al. [5] introduced LiDAR snowfall simulation techniques to augment training data, demonstrating improved detection robustness through synthetic weather augmentation.

Domain adaptation techniques have been explored to bridge the distribution gap between clear and adverse weather conditions. WEDGE [27] constructed a multi-weather autonomous driving dataset using generative vision-language models, facilitating training under diverse weather conditions including fog, rain, and snow. Rothmeier and Huber [28] developed standardized test methodologies for evaluating vision-based detection algorithms under adverse conditions, while Hasirlioglu et al. [29] proposed sensor performance evaluation frameworks specific to rain scenarios.

Despite these advances, existing approaches face fundamental limitations when dealing with extreme degradation. Statistical filtering methods can remove weather-induced noise but cannot recover lost geometric information when point cloud density falls below critical thresholds. Domain adaptation relies on sufficient feature preservation in degraded images, which breaks down when visibility approaches sensor physical limits. These limitations motivate the exploration of alternative sensing modalities that maintain information content under severe weather conditions.

From a broader machine learning perspective, perception under extreme weather is also closely related to rare-event prediction in imbalanced, high-risk environments. Abdulrazaq [30] highlights that, in such scenarios, model development should be accompanied by evaluation protocols that emphasize minority and failure-critical cases, including precision–recall-oriented analysis and robustness-focused assessment rather than overall accuracy. This viewpoint aligns with our focus on adverse weather breakdown regimes, where the primary challenge is not average-condition performance but reliable behavior under low-frequency, high-consequence sensing failures.

### 2.3. Gated Imaging for Autonomous Driving

Gated imaging technology exploits the time-of-flight principle to achieve range-gated sensing, effectively suppressing backscattered light from atmospheric particles. The fundamental principle involves synchronizing a pulsed light source with a camera shutter operating at nanosecond precision, enabling selective capture of photons from specific distance ranges [2]. This temporal discrimination capability provides inherent robustness to volumetric scattering phenomena such as fog and rain.

Bijelic et al. [2] demonstrated the first deep learning framework for gated imaging in adverse weather, showing that gated images preserve structural features even in dense fog with visibility below 50 m. Their multi-modal fusion network combined gated, RGB, and LiDAR inputs, achieving significant improvements over conventional sensors in synthetic and real foggy conditions. Gruber et al. [31] introduced Gated2Depth, a method to reconstruct dense depth maps from gated image sequences by leveraging the implicit range information encoded in gated acquisitions. Their approach employed adversarial training to handle the domain shift between gated and conventional imaging.

Subsequent work by Walia et al. [11] proposed self-supervised depth estimation from gated images without requiring LiDAR ground truth, using temporal consistency across multiple gated slices as the supervisory signal. This advancement reduced the data annotation burden for training gated image processing networks. Laurenzis et al. [32] developed physics-based models for gated imaging in scattering media, providing theoretical foundations for understanding backscatter suppression as a function of gate timing parameters.

Despite these advances, several challenges hinder the widespread adoption of gated imaging in autonomous vehicles. First, active illumination requirements limit effective operating range and introduce potential interference in multi-vehicle scenarios. Second, gated cameras typically acquire depth slices sequentially, requiring either multiple gates per frame or sophisticated depth reconstruction algorithms. Third, gated imaging provides minimal advantage over conventional cameras in clear weather, motivating the need for adaptive fusion strategies that dynamically leverage gated images only when environmental conditions warrant their use.

Our work builds upon these foundations by introducing an adaptive fusion framework that treats gated imaging and LiDAR as complementary modalities with weather-dependent reliability. Unlike prior approaches that use fixed fusion weights [2] or modality-specific networks without cross-modal adaptation, we propose a unified architecture that learns to assess per-modality confidence and dynamically adjusts fusion weights based on real-time feature quality assessment.

## 3. Preliminaries

This section establishes the theoretical foundations for our approach, covering the physical principles of gated imaging and the formal problem formulation for adaptive multi-modal fusion.

### 3.1. Gated Imaging Principles

Gated imaging exploits the time-of-flight (ToF) principle to achieve range-selective sensing. The system comprises a pulsed illumination source (typically a near-infrared laser) synchronized with a camera equipped with a fast electronic shutter. The operational sequence proceeds as follows:

1. A laser pulse of duration $\tau_{p}\approx 10$ ns illuminates the scene at time $t_{0}$.
2. Photons propagate to objects at distance *d*, requiring time $t_{direct}=2d/c$ for the round trip, where *c* is the speed of light.
3. Scattered photons from atmospheric particles travel longer optical paths, arriving with delays $t_{scatter}>t_{direct}$.
4. The camera shutter opens at time $t_{gate}=t_{0}+t_{delay}$ for duration $\tau_{g}\approx 10$ ns, capturing only photons from the range slice $\left[d_{min},d_{max}\right]$.

The range selectivity is determined by

(1) $$d_{min}=\frac{c\cdot t_{delay}}{2},\;d_{max}=\frac{c\cdot (t_{delay}+\tau_{g})}{2}.$$

This temporal gating effectively filters backscatter because multiply scattered photons from nearby particles accumulate additional optical path length, causing them to arrive outside the narrow temporal window. The benefit of gating can be expressed in terms of the signal-to-backscatter ratio (SBR). For a continuous-wave camera, backscatter is integrated over the full exposure time $\tau_{exposure}$ (on the order of milliseconds), whereas a gated camera integrates backscatter only over the short gate window $\tau_{g}$ (on the order of nanoseconds) that brackets the target range. Because the in-gate signal energy from the target is approximately preserved while the integrated backscatter scales with the integration window, the relative backscatter contribution is reduced by a factor of approximately $\tau_{g}/\tau_{exposure}$, so that

(2) $${\mathrm{SBR}}_{gated}\approx \frac{\tau_{exposure}}{\tau_{g}}\cdot {\mathrm{SBR}}_{conventional},$$

i.e., the gated SBR improves over the conventional SBR by the ratio $\tau_{exposure}/\tau_{g}\gg 1$. With $\tau_{exposure}$ in the millisecond range and $\tau_{g}\approx 10$ ns, this improvement factor can exceed two orders of magnitude in dense fog, consistent with the empirical observations reported in [2]. We note that the previous version of this manuscript stated this ratio with the factor inverted; the corrected form above is the one that yields the claimed improvement.

### 3.2. Problem Formulation

We formulate the adaptive multi-modal fusion problem as follows. Let $G=\{G_{1},G_{2},\ldots ,G_{n}\}$ represent a sequence of gated images captured at different range gates, and $P={\left\{p_{i}\right\}}_{i=1}^{N}$ denote the LiDAR point cloud with *N* points, where each point $p_{i}=\left(x_{i},y_{i},z_{i},r_{i}\right)$ contains 3D coordinates and reflectance intensity. The goal is to detect a set of 3D bounding boxes $B={\left\{b_{j}\right\}}_{j=1}^{M}$, where each box $b_{j}=\left(x,y,z,l,w,h,\theta ,c\right)$ specifies the object’s center position, dimensions, orientation, and class.

The key challenge is that sensor reliability varies with environmental conditions. Let $\eta_{g}\left(t\right)$ and $\eta_{l}\left(t\right)$ denote the time-varying reliability (information content) of gated imaging and LiDAR modalities at time *t*. Under clear conditions, $\eta_{g}\approx \eta_{l}$, but in dense fog, the reliability of conventional RGB imaging collapses ($\eta_{rgb}\ll \eta_{l}$) while gated imaging maintains high $\eta_{g}$. An optimal fusion strategy should satisfy

(3) $$F^{*}\left(G,P\right)=\arg\max_{F}E\left[\mathrm{AP}\left(F\left(G,P\right),B_{gt}\right)|\eta_{g}\left(t\right),\eta_{l}\left(t\right)\right],$$

where AP denotes average precision and $B_{gt}$ are ground-truth annotations.

Our approach models this through learned adaptive weights $w_{g},w_{l}\in \left[0,1\right]$ such that $w_{g}+w_{l}=1$, where the weights automatically adjust based on implicit estimation of $\eta_{g}$ and $\eta_{l}$ from feature-level uncertainty. This formulation avoids requiring explicit weather condition labels during inference while enabling smooth transitions between modality-dominant regimes.

### 3.3. Feature Space Alignment

A critical challenge in fusing gated images and LiDAR point clouds lies in their disparate data structures: images are dense 2D grids while point clouds are sparse 3D irregular sets. We establish correspondence through perspective projection. Let a 3D point be expressed in homogeneous LiDAR coordinates as $X={\left[x,y,z,1\right]}^{T}$ and its image projection in homogeneous pixel coordinates as $\tilde{x}={\left[u,v,1\right]}^{T}$. The projection is

(4) $$s\;{\left[u,v,1\right]}^{T}=K\;\left[R|t\right]\;{\left[x,y,z,1\right]}^{T},$$

where $K\in R^{3\times 3}$ is the camera intrinsic matrix, $\left[R|t\right]\in R^{3\times 4}$ is the extrinsic transformation (rotation R∈SO(3) and translation $t\in R^{3}$) from LiDAR to camera coordinates, and s>0 is the perspective scale factor equal to the point depth in the camera frame. The pixel coordinates are recovered as $u={\left(K\left[R|t\right]X\right)}_{1}/s$ and $v={\left(K\left[R|t\right]X\right)}_{2}/s$ after dividing by *s*. This projection enables: (1) augmenting each LiDAR point with gated image features at pixel (u,v), and (2) projecting image features into 3D space for volumetric fusion. Our architecture exploits both directions to maximize cross-modal information exchange.

## 4. Methodology

This section presents the Adaptive Feature-Weighting Network (AFW-Net), a novel multi-modal fusion framework that dynamically adjusts sensor contributions based on environmental degradation. The architecture comprises three core components: a cross-modal feature extraction module that processes gated images and LiDAR point clouds through specialized encoders, an attention-based adaptive fusion mechanism that estimates per-modality reliability and computes fusion weights, and a degradation-aware training strategy that enables the network to learn weather-dependent fusion policies without explicit condition labels.

### 4.1. Architecture Overview

**Figure 1:**
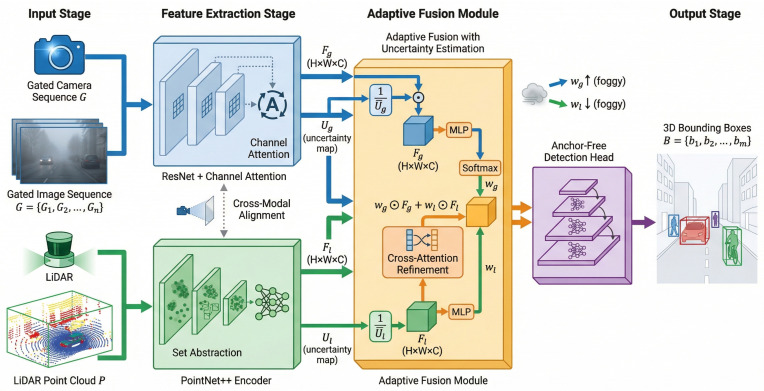
Overall architecture of the proposed Adaptive Feature-Weighting Network (AFW-Net).

The AFW-Net architecture follows a single-stage, anchor-free detection paradigm built on dual modality-specific encoders followed by an adaptive fusion module, as shown in Figure 1. Given a gated image sequence G and LiDAR point cloud P, the network first extracts modality-specific features through dedicated encoders, then fuses these features through an adaptive weighting module that dynamically adjusts contributions based on estimated feature quality. The fused representation feeds into a detection head that predicts 3D bounding boxes with associated confidence scores. We emphasize that the detection head is single-stage and anchor-free (FCOS-style, Section 4.4); the network does not employ a separate region-proposal stage. The earlier description of a “two-stage” paradigm was inaccurate and has been corrected throughout.

Let $F_{g}=\phi_{g}\left(G;\theta_{g}\right)$ and $F_{l}=\phi_{l}\left(P;\theta_{l}\right)$ denote the gated image and LiDAR features extracted by encoders $\phi_{g}$ and $\phi_{l}$ with parameters $\theta_{g}$ and $\theta_{l}$ respectively. At the fusion stage both feature maps share a common spatial resolution and channel dimension, $F_{g},{\tilde{F}}_{l}\in R^{H\times W\times C}$, as detailed in Section 4.3 (Common Feature Space). The adaptive fusion module Ψ computes reliability-weighted features:

(5) $$F_{fused}=\Psi \left(F_{g},{\tilde{F}}_{l};\theta_{\Psi }\right)=w_{g}⊙F_{g}+w_{l}⊙{\tilde{F}}_{l},$$

where $w_{g},w_{l}\in R^{C}$ are channel-wise adaptive weights learned through attention mechanisms, and ⊙ denotes element-wise multiplication with broadcasting. The key innovation lies in computing these weights dynamically based on feature uncertainty rather than using fixed values across all conditions.

### 4.2. Cross-Modal Feature Extraction

The gated image encoder exploits both intensity and implicit depth information encoded in gated acquisitions. Since gated images capture range slices corresponding to specific distance intervals, the sequence $G=\{G_{1},\ldots ,G_{n}\}$ contains complementary depth-selective views of the scene. We employ a ResNet-based encoder with channel attention modules to process this sequence. Each gated image $G_{i}$ is processed through convolutional layers with batch normalization

(6) $$h_{i}=\mathrm{ReLU}\left(\mathrm{BN}\left(\mathrm{Conv}\left(G_{i}\right)\right)\right),$$

where the convolutional operator extracts hierarchical features at multiple scales. The channel attention mechanism reweights feature channels based on their informativeness

(7) $$\alpha_{c}=\sigma \left(\mathrm{MLP}\left(\mathrm{GAP}\left(h_{i}\right)\right)\right),$$

where GAP denotes global average pooling, MLP is a two-layer perceptron, and σ is the sigmoid activation. The attention-weighted features from all gates are aggregated through max-pooling across the gate dimension, producing a unified gated feature representation $F_{g}\in R^{H\times W\times C}$.

For LiDAR point cloud processing, we adopt a PointNet++ architecture that captures hierarchical geometric features while maintaining permutation invariance. The point cloud P undergoes set abstraction layers that progressively downsample points while expanding receptive fields:

(8) $$P^{\left(l+1\right)}=\mathrm{SA}\left(P^{\left(l\right)};\theta_{sa}^{\left(l\right)}\right),$$

where each set abstraction layer groups neighboring points within radius *r*, applies a shared MLP to local neighborhoods, and performs max-pooling to obtain aggregated features. This hierarchical structure captures both local geometric patterns and global contextual information. The resulting point features are voxelized into a 3D grid representation $F_{l}\in R^{D\times H\times W\times C}$ (depth *D*, height *H*, width *W*, channels *C*) to enable efficient convolutional processing in subsequent stages.

Cross-modal alignment is achieved through geometric projection as established in Section 3. For each LiDAR point $p_{i}$, we project its coordinates to the gated image plane using the calibrated transformation, retrieving the corresponding gated feature vector through bilinear interpolation. This augments each point with appearance information

(9) $$p_{i}^{aug}=\left[p_{i};F_{g}\left(u_{i},v_{i}\right)\right],$$

where $\left(u_{i},v_{i}\right)$ are the projected pixel coordinates and $F_{g}\left(u_{i},v_{i}\right)$ denotes the bilinearly interpolated feature. This bidirectional information flow ensures that geometric LiDAR features are enriched with appearance cues from gated imaging, while gated features benefit from precise geometric context.

For clarity, we summarize the feature dimensions used throughout the pipeline. The gated encoder outputs $F_{g}\in R^{H\times W\times C}$ with an associated uncertainty map $U_{g}\in R^{H\times W\times C}$. The LiDAR encoder outputs voxel features $F_{l}\in R^{D\times H\times W\times C}$, which are collapsed along the depth axis *D* by max-pooling to ${\tilde{F}}_{l}\in R^{H\times W\times C}$ with uncertainty map $U_{l}\in R^{H\times W\times C}$ before fusion. Thus all quantities entering Equation (5) share the dimension H×W×C, and the channel-wise weights satisfy $w_{g},w_{l}\in R^{C}$. In all experiments C=256 and (H,W) corresponds to the stride-8 feature pyramid level.

### 4.3. Adaptive Fusion with Uncertainty Estimation

The core innovation of AFW-Net lies in its adaptive fusion mechanism that dynamically adjusts modality weights based on estimated feature reliability. Unlike fixed-weight fusion that treats all sensors equally regardless of degradation, our approach quantifies per-modality uncertainty and redistributes reliance accordingly.

We model feature uncertainty through learned variance estimation. Following the heteroscedastic aleatoric-uncertainty formulation common in Bayesian deep learning, each modality branch predicts not only a feature map but also a spatially varying variance that captures how reliable those features are; the variance is then used both as a supervision target (Section 4.4) and as the signal that drives fusion weighting. We adopt this learned-variance formulation, rather than a fixed heuristic such as feature magnitude or entropy, because it can be trained end-to-end against a concrete clean-feature reference and yields a per-channel reliability estimate that is directly comparable across modalities. For each modality, the encoder produces both a feature map and an associated uncertainty map. Specifically, the gated encoder outputs $\left(F_{g},U_{g}\right)$ where $U_{g}\in R^{H\times W\times C}$ estimates per-channel uncertainty, computed through an auxiliary branch:

(10) $$U_{g}=\mathrm{softplus}\left({\mathrm{Conv}}_{1\times 1}\left(F_{g}\right)\right).$$

The softplus activation ensures non-negative uncertainty values. Similarly, the LiDAR encoder produces $\left({\tilde{F}}_{l},U_{l}\right)$. Higher uncertainty indicates degraded or unreliable features, which should receive lower weights in the fusion process.

The adaptive weights are computed through an attention mechanism that considers both feature statistics and uncertainty estimates. We first compute global context vectors through spatial pooling:

(11) $$c_{g}=\frac{1}{HW}\sum_{i,j}F_{g}\left(i,j\right),\;c_{l}=\frac{1}{HW}\sum_{i,j}{\tilde{F}}_{l}\left(i,j\right).$$

The reliability scores are then derived by combining feature magnitude with inverse uncertainty:

(12) $$s_{g}={\mathrm{MLP}}_{w}\left(\left[c_{g};1/{\bar{U}}_{g}\right]\right),\;s_{l}={\mathrm{MLP}}_{w}\left(\left[c_{l};1/{\bar{U}}_{l}\right]\right),$$

where ${\bar{U}}_{g}$ and ${\bar{U}}_{l}$ denote spatially averaged uncertainty, and [·;·] indicates concatenation. The MLP networks learn to map these combined statistics to reliability scores. Finally, normalized weights are obtained through softmax:

(13) $$w_{g}=\frac{\exp(s_{g})}{\exp\left(s_{g}\right)+\exp\left(s_{l}\right)},\;w_{l}=\frac{\exp(s_{l})}{\exp\left(s_{g}\right)+\exp\left(s_{l}\right)}.$$

This formulation ensures that weights sum to unity and smoothly transition as relative reliability changes. When gated imaging encounters degradation (high $U_{g}$), the weight $w_{g}$ automatically decreases, shifting reliance toward LiDAR. Conversely, in dense fog where LiDAR uncertainty increases while gated imaging remains reliable, the system emphasizes gated features.

The fused features undergo further refinement through cross-modal attention layers that enable interaction between modalities:

(14) $$\begin{array}{cc} F_{refined} & =F_{fused}\\ & +\beta \cdot \mathrm{Attention}(Q=F_{fused},K=F_{g},V={\tilde{F}}_{l}), \end{array}$$

where β is a learnable scaling parameter. This attention mechanism allows the fused representation to selectively retrieve complementary information from individual modality features, enhancing robustness beyond simple weighted averaging.

**Common Feature Space**. Here, we would like to clarify that to enable meaningful fusion, the modality-specific features $F_{g}$ and ${\tilde{F}}_{l}$ must reside in a shared representational space with compatible dimensionality and semantic alignment. We achieve this through three mechanisms. First, both encoders are designed to produce feature maps of identical spatial resolution H×W and channel dimension *C* at the fusion stage: the gated encoder outputs $F_{g}\in R^{H\times W\times C}$, while the LiDAR encoder’s voxel features $F_{l}\in R^{D\times H\times W\times C}$ are collapsed along the depth axis via max-pooling to yield ${\tilde{F}}_{l}\in R^{H\times W\times C}$. Second, a shared projection layer (implemented as a 1×1 convolution followed by normalization) is applied to each modality to map features into a common embedding space. Third, the cross-modal attention refinement described in Equation (14) further promotes alignment by enabling information exchange between modalities. This design ensures that the element-wise fusion in Equation (5) operates on semantically compatible representations.

### 4.4. Detection Head and Loss Functions

The refined features $F_{refined}$ feed into a detection head based on the anchor-free FCOS paradigm. Each spatial location in the feature map predicts a 3D bounding box, objectness score, and classification logits. The box parameterization follows:

(15) $$\hat{b}=\left(x,y,z,l,w,h,\sin\theta ,\cos\theta ,c\right),$$

where orientation is encoded as sine and cosine to avoid discontinuity issues.

The training objective combines three loss terms:

(16) $$L_{total}=L_{cls}+\lambda_{reg}L_{reg}+\lambda_{unc}L_{unc}.$$

The classification loss employs focal loss to address class imbalance:

(17) $$L_{cls}=-\frac{1}{N_{pos}}\sum_{i}\alpha_{i}{\left(1-p_{i}\right)}^{\gamma }\log\left(p_{i}\right),$$

where $\alpha_{i}$ balances foreground and background, and γ=2 focuses learning on hard examples.

The regression loss combines smooth L1 loss for box parameters with IoU-based loss for better localization:

(18) $$L_{reg}=\sum_{i}I_{pos}\left(i\right)\left[\mathrm{SmoothL}1\left({\hat{b}}_{i},b_{i}\right)+\left(1-\mathrm{IoU}\left({\hat{b}}_{i},b_{i}\right)\right)\right].$$

The uncertainty regularization loss supervises the per-modality uncertainty maps so that they reflect feature reliability under degradation. It penalizes the negative log-likelihood under a Gaussian (heteroscedastic) assumption:

(19) $$L_{unc}=\sum_{m\in \{g,l\}}\left[\frac{∥{\hat{F}}_{m}-F_{m}^{gt}{∥}^{2}}{2U_{m}^{2}}+\frac{1}{2}\log U_{m}^{2}\right],$$

where ${\hat{F}}_{m}$ is the student feature predicted by the AFW-Net encoder for modality *m*, $U_{m}$ is its predicted uncertainty, and $F_{m}^{gt}$ represents per-modality feature targets obtained through teacher–student distillation rather than from box-level labels. Specifically, we pre-train a single-modality teacher network on clean-weather data for each modality (a ResNet-50 gated image detector and a PointNet++ LiDAR detector). The feature maps produced by these teacher networks on clean inputs serve as $F_{m}^{gt}$—the “ideal” features that the student encoders within AFW-Net should approximate. During training, the teacher weights are frozen, and the student encoders are trained to both match these targets and estimate calibrated uncertainty $U_{m}$. The mechanism by which this drives adaptive weighting is as follows: when a modality is degraded (e.g., LiDAR in fog), its student features ${\hat{F}}_{m}$ deviate from the clean teacher targets $F_{m}^{gt}$; minimizing the first term of $L_{unc}$ then forces the predicted $U_{m}$ to grow so as to down-weight the now-unreliable squared-error term, while the second term $\frac{1}{2}\log U_{m}^{2}$ prevents $U_{m}$ from growing without bound. A larger $U_{m}$ in turn lowers the reliability score $s_{m}$ through the $1/{\bar{U}}_{m}$ term in Equation (13) and therefore reduces the fusion weight for that modality via the softmax in Equation (14). In this way the uncertainty supervision and the fusion weighting are explicitly coupled. This loss balances prediction accuracy with uncertainty magnitude, preventing the network from trivially minimizing error by reporting infinite uncertainty.

### 4.5. Degradation-Aware Training Strategy

To enable the network to learn weather-dependent fusion without explicit condition labels, we employ a degradation-aware training strategy with synthetic weather augmentation. During training, we randomly apply fog simulation to gated images and raindrop simulation to LiDAR point clouds with varying severity levels.

Fog augmentation for gated images follows the atmospheric scattering model:

(20) $$G_{fog}\left(x\right)=G\left(x\right)\cdot e^{-\beta d(x)}+L_{\infty }\left(1-e^{-\beta d(x)}\right),$$

where β is the scattering coefficient, d(x) is the depth at pixel *x*, and $L_{\infty }$ is the atmospheric light. The scattering coefficient β is sampled from a range corresponding to visibility from 30 m to 200 m, creating diverse degradation levels.

LiDAR augmentation simulates precipitation effects through point dropout and noise injection. Points are randomly removed with probability proportional to their distance to simulate beam attenuation:

(21) $$P_{drop}\left(p_{i}\right)=1-e^{-\alpha ∥p_{i}∥}.$$

Additionally, random points are injected near the sensor to simulate raindrop reflections, with density controlled by augmentation severity.

The key insight is that by training with correlated degradation between modalities, the network learns to implicitly recognize degradation patterns through feature statistics. When real-world fog reduces gated image quality, the learned uncertainty estimator produces higher $U_{g}$ values, automatically reducing $w_{g}$. This eliminates the need for explicit weather condition classification during inference.

The complete training procedure alternates between clean and augmented samples with balanced sampling to prevent bias toward either extreme. We employ curriculum learning by gradually increasing augmentation severity over training epochs, allowing the network to first learn robust features on clean data before adapting to degraded conditions.

### 4.6. Key Assumptions and Limitations

Our framework rests on several assumptions that bound its applicability. First, we assume that the extrinsic calibration between the gated camera and LiDAR remains approximately constant during operation; significant calibration drift would degrade cross-modal alignment (see Section 5.5 for quantitative analysis). Second, the atmospheric scattering model used for fog augmentation (Equation (20)) assumes homogeneous fog, which may not fully capture real-world patchy or layered fog distributions. Third, the uncertainty estimation is trained on synthetic degradation patterns; while the learned estimator generalizes well to real fog (as demonstrated empirically), its behavior under conditions substantially different from training—such as heavy rain, snow, sandstorms or volcanic ash—remains outside the scope of the present experimental validation. Fourth, we assume that gated and LiDAR modalities provide complementary rather than redundant information; in rare scenarios where both modalities fail simultaneously (e.g., extremely dense fog beyond 20 m visibility combined with heavy rain), the framework cannot recover lost information. These assumptions are revisited in the limitations discussion in Section 6.

### 4.7. Inference Procedure

Algorithm 1 summarizes the complete inference pipeline. The computational flow emphasizes the automatic weight adjustment without requiring manual intervention or weather sensing.
**Algorithm 1** AFW-Net Inference**Require:** Gated image sequence G, LiDAR point cloud P**Ensure:** 3D bounding boxes B 1:$\left(F_{g},U_{g}\right)\leftarrow \phi_{g}\left(G\right)$ {Gated feature extraction} 2:$\left({\tilde{F}}_{l},U_{l}\right)\leftarrow \phi_{l}\left(P\right)$ {LiDAR feature extraction} 3:Align ${\tilde{F}}_{l}$ and $F_{g}$ via camera-LiDAR projection 4:$c_{g}\leftarrow \mathrm{GlobalPool}\left(F_{g}\right)$, $c_{l}\leftarrow \mathrm{GlobalPool}\left({\tilde{F}}_{l}\right)$ 5:$s_{g}\leftarrow {\mathrm{MLP}}_{w}\left(\left[c_{g};1/\mathrm{mean}\left(U_{g}\right)\right]\right)$ 6:$s_{l}\leftarrow {\mathrm{MLP}}_{w}\left(\left[c_{l};1/\mathrm{mean}\left(U_{l}\right)\right]\right)$ 7:$w_{g},w_{l}\leftarrow \mathrm{Softmax}\left(\left[s_{g},s_{l}\right]\right)$ {Adaptive weights} 8:$F_{fused}\leftarrow w_{g}⊙F_{g}+w_{l}⊙{\tilde{F}}_{l}$ 9:$F_{refined}\leftarrow F_{fused}+\mathrm{CrossAttention}\left(F_{fused},F_{g},{\tilde{F}}_{l}\right)$10:$B\leftarrow \mathrm{DetectionHead}(F_{refined})$ {Predict boxes}11:**return** B

The algorithm highlights the seamless integration of uncertainty-based weight computation within the forward pass, requiring no additional computational overhead beyond the dual-branch encoders. The adaptive mechanism operates entirely through learned parameters, maintaining real-time inference capability essential for autonomous driving applications.

## 5. Experiments

This section presents comprehensive experimental evaluation of the proposed AFW-Net framework. We first describe the dataset, evaluation metrics, and experimental setup, then present comparative results against state-of-the-art baselines, followed by ablation studies analyzing each component’s contribution, and conclude with qualitative analysis and discussion.

### 5.1. Dataset and Evaluation Protocol

**Princeton Automated Driving Dataset.** We conduct experiments on the Princeton Automated Driving Dataset [2], which provides synchronized gated imaging, LiDAR, and RGB camera data captured under diverse weather conditions. The dataset comprises 15,000 frames collected across clear, foggy, and rainy scenarios in both daytime and nighttime conditions. Each frame includes: (1) a sequence of three gated images captured at different range gates (10–30 m, 30–50 m, 50–80 m), (2) Velodyne HDL-64E LiDAR point clouds with approximately 120,000 points per frame, (3) conventional RGB images for comparison, and (4) 3D bounding box annotations for vehicles, pedestrians, and cyclists. The dataset exhibits significant environmental diversity, with fog density ranging from clear conditions (200 m visibility) to dense fog (30–50 m visibility), measured using meteorological optical range metrics.

**Fog Visibility Quantification.** Fog severity is quantified by the meteorological optical range (MOR), defined as the distance at which the contrast of a collimated light beam falls to 5% of its original value. For real captures, MOR is obtained from the visibility/transmissometer readings provided with the Princeton dataset; for synthetically fogged samples, the target MOR is set directly through the scattering coefficient β in the atmospheric model of Equation (20), using the standard Koschmieder relation MOR≈3.0/β. We bin conditions into clear (MOR>100 m), moderate fog (50–100 m), and dense fog (<50 m) following these readings.

**Label Assignment and Validation.** The 3D bounding box annotations are those released with the Princeton dataset and are reused without modification. We did not relabel the data. To verify label integrity after our preprocessing and coordinate transforms, two of the authors independently spot-checked a random 5% subset (750 frames) by overlaying the provided boxes on both the LiDAR point cloud and the gated images; frames with projection mismatches were excluded. Synthetic augmentation (fog/dropout) is applied only to the sensor inputs and never alters the ground-truth boxes, so label correctness is preserved by construction.

**Dataset Limitations.** We note several limitations. (i) The dataset emphasizes fog and clear conditions; heavy rain and snow frames are comparatively scarce, which constrains the conclusions we can draw for those conditions (see Section 6). (ii) Real dense fog captures are inherently limited in number, so part of the dense fog training distribution is synthetic. (iii) The dataset is geographically concentrated, motivating the cross-dataset evaluation in Section 5.8. (iv) Class distribution is dominated by vehicles, with fewer pedestrian and cyclist instances, which increases variance in the minority-class metrics.

We partition the dataset into training (10,500 frames), validation (1500 frames), and testing (3000 frames) sets, ensuring balanced weather condition distribution across splits. Crucially, the test set includes weather severity levels not seen during training to evaluate generalization capability. Following standard autonomous driving evaluation protocols, we categorize detections by object distance: easy (0–30 m), moderate (30–50 m), and hard (50–80 m), reflecting the increasing difficulty at longer ranges, particularly under adverse weather.

**Data Split Details.** The training set contains approximately 5250 clear-weather frames, 3150 moderate fog frames, and 2100 dense fog frames. The validation set maintains the same 50%/30%/20% ratio across weather conditions. The test set is partitioned as 1200 clear, 900 moderate fog, and 900 dense fog frames, with fog density levels sampled to include conditions not present in training (e.g., visibility between 35 and 45 m that fills the gap between training fog categories). All splits are geographically disjoint—frames from the same driving route appear in only one split.

**Evaluation Metrics.** We adopt average precision (AP) at IoU threshold 0.7 for vehicles and 0.5 for pedestrians and cyclists as the primary metric, consistent with KITTI benchmark conventions [33]. Additionally, we report Average Orientation Similarity (AOS) to assess orientation estimation accuracy, and recall at various distance thresholds. To analyze weather-specific performance, we compute AP separately for three visibility conditions: clear (>100 m), moderate fog (50–100 m), and dense fog (<50 m). The mean average precision (mAP) reported in Table 1 is computed as the arithmetic mean of the vehicle-class AP values across the three weather conditions (clear, moderate fog, dense fog); i.e., it is a within-class average over weather conditions and not an average across object classes, providing an overall performance indicator that weights each condition equally.

### 5.2. Implementation Details

The gated encoder employs ResNet-50 as the backbone with channel attention modules inserted after each residual block. The LiDAR encoder uses PointNet++ [16] with four set abstraction layers, with sampling radii of [0.2 m, 0.4 m, 0.8 m, 1.6 m] and group sizes of [32, 64, 128, 256]. The adaptive fusion module consists of two-layer MLPs with hidden dimension 256 for weight computation. The detection head follows the FCOS architecture with feature pyramid levels at strides [8, 16, 32, 64, 128] pixels.

Training employs the Adam optimizer with initial learning rate $3\times 10^{-4}$, decayed by a factor of 0.1 at epochs 80 and 110, for a total of 120 epochs. The batch size is set to 16 on 4 NVIDIA RTX 3090 GPUs. Loss weights are $\lambda_{reg}=2.0$ and $\lambda_{unc}=0.5$. Data augmentation includes random horizontal flipping, rotation (±10 degrees), and scaling (0.95–1.05). The degradation-aware augmentation applies fog with β∈[0.01,0.15] and LiDAR dropout with rates ∈[0.1,0.4] to 60% of training samples, with curriculum scheduling gradually increasing augmentation probability from 0.3 to 0.6 over the first 40 epochs.

All experiments are implemented in PyTorch 1.13. Inference processes approximately 12 frames per second on a single RTX 3090 GPU, satisfying real-time requirements for autonomous driving applications.

**Baseline Reproduction.** All RGB-LiDAR baselines (PointPainting, MV3D, 3D-CVF) are retrained from scratch on our training split using the official open-source implementations with default hyperparameters, substituting the gated image input with RGB images to ensure a fair comparison on the same data. Single-modality baselines (PointPillars, CenterNet) are likewise trained on the same split. The fixed-weight gated-LiDAR baseline follows the architecture of Bijelic et al. [2] with equal fusion weights ($w_{g}=w_{l}=0.5$), re-implemented and trained under identical conditions. All methods use the same train/val/test splits and evaluation code.

### 5.3. Baseline Comparisons

We compare AFW-Net against state-of-the-art multi-modal fusion methods for 3D object detection:

**Single-Modality Baselines:** PointPillars [22] (LiDAR-only) and CenterNet with ResNet-50 (image-only) establish performance bounds for individual sensors.

**RGB-LiDAR Fusion:** PointPainting [7] augments LiDAR points with RGB semantic features; MV3D [17] fuses multi-view representations; 3D-CVF [24] employs cross-view spatial fusion.

**Gated-LiDAR Fusion:** We implement the baseline from Bijelic et al. [2] using fixed-weight fusion of gated and LiDAR features as a direct comparison to our adaptive approach.

Table 1 presents the quantitative comparison across different weather conditions. Under clear conditions, all multi-modal methods achieve comparable performance, with AFW-Net obtaining 89.7% vehicle AP, slightly outperforming fixed-weight gated-LiDAR fusion (88.9%) due to the adaptive mechanism’s ability to suppress noise even in favorable conditions. The performance gap becomes pronounced under moderate fog, where AFW-Net maintains 85.3% vehicle AP while RGB-LiDAR methods degrade to 72.1–76.8%. Most critically, under dense fog conditions where visibility drops below 50 m, AFW-Net achieves 82.1% vehicle AP, representing a 23.7% relative improvement over the best RGB-LiDAR baseline (PointPainting: 58.4%) and 7.3% improvement over fixed-weight gated-LiDAR fusion (74.8%).

Table 2 reports class-wise and distance-wise results on the dense fog test set. AFW-Net improves over both baselines for all three classes and at all three distance bins. The gains are largest in the most challenging hard (50–80 m) bin, where AFW-Net reaches 66.7% vehicle AP versus 39.4% for PointPainting, and similarly improves pedestrian and cyclist AP in this range. Absolute AP for pedestrians and cyclists is lower than for vehicles, reflecting both their smaller physical size and their lower instance counts in the dataset (Section 5.1, Dataset Limitations); nonetheless the relative ordering of methods is preserved across classes.

The standard deviations across random seeds demonstrate the robustness of our method, with AFW-Net exhibiting lower variance (±0.2–0.5%) compared to RGB-LiDAR baselines (±0.9–1.8% under fog), indicating more stable performance in challenging conditions. This stability stems from the adaptive mechanism’s consistent uncertainty estimation across different random initializations. To assess whether the improvements are statistically meaningful, we conducted paired two-sided *t*-tests over the per-seed, per-frame AP values comparing AFW-Net against the strongest baseline in each condition. The improvement over fixed-weight gated-LiDAR fusion under dense fog (82.1 vs. 74.8) is significant at p<0.01, and the improvement over PointPainting under dense fog is significant at p<0.001; the difference under clear conditions (89.7 vs. 88.9) is not significant at the 0.05 level, consistent with the expectation that adaptivity matters most under degradation.

**Figure 2:**
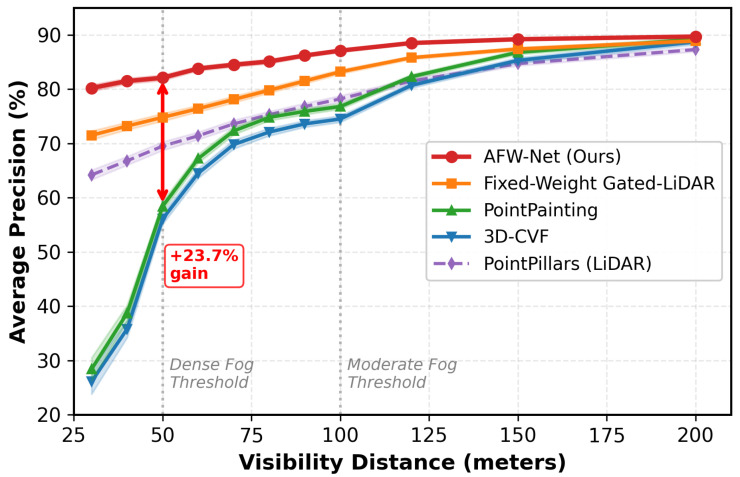
Detection AP as a function of visibility distance for different methods. AFW-Net maintains robust performance even as visibility decreases below 50 m, while RGB-LiDAR fusion methods exhibit catastrophic degradation. The adaptive fusion provides more graceful performance decline compared to fixed-weight fusion, particularly in the critical 30–70 m visibility range. The shaded bands around each curve indicate the variation range of AP across repeated evaluations with different seeds.

Figure 2 illustrates performance degradation as a function of visibility distance. While RGB-LiDAR methods exhibit exponential performance drop below 80 m visibility, AFW-Net maintains relatively graceful degradation with a gentler slope. The fixed-weight gated-LiDAR baseline shows improvement over RGB-LiDAR but still experiences notable degradation under extreme conditions, whereas AFW-Net’s adaptive weighting provides additional resilience.

### 5.4. Ablation Studies

We conduct systematic ablation studies to validate each component’s contribution. Table 3 presents results on the dense fog test set where component effects are most pronounced. All ablation AP values are vehicle-class AP at IoU 0.7 on the dense fog (<50 m) test split, averaged over three seeds.

**Uncertainty Estimation.** Removing uncertainty estimation (using only feature magnitude for weight computation) reduces performance by 5.8%, demonstrating that explicit uncertainty modeling is crucial for assessing modality reliability. Without uncertainty, the network cannot effectively distinguish between strong features from reliable sensors versus spurious high-magnitude activations from degraded inputs.

**Cross-Modal Attention.** Ablating the cross-attention refinement decreases AP by 3.2%. While the primary fusion through weighted averaging provides substantial benefits, the attention mechanism enables fine-grained feature exchange that recovers complementary details. Visualization reveals that cross-attention selectively retrieves geometric boundaries from LiDAR when gated image edges are blurred by residual scatter.

**Degradation-Aware Augmentation.** Training without weather-specific augmentation severely impacts performance (7.6% drop), validating our hypothesis that exposure to diverse degradation patterns during training enables better uncertainty calibration. Interestingly, this ablation shows higher variance (±1.2%) across random seeds, suggesting that without augmentation, the network’s behavior under extreme conditions becomes less predictable.

**Adaptive vs. Fixed Weights.** Replacing adaptive weights with fixed $w_{g}=w_{l}=0.5$ reduces AP by 7.3%, directly demonstrating the value of dynamic adjustment. This comparison isolates the contribution of adaptivity from the benefits of using gated imaging itself.

**Gated vs. RGB under the Same Adaptive Architecture.** To isolate how much of the gain stems from the gated sensing modality itself versus the generic adaptive fusion architecture, we additionally trained the identical AFW-Net architecture (same encoders, uncertainty branches, adaptive weighting, degradation-aware training, and detection head) with the gated input replaced by the conventional RGB stream, yielding an “AFW-Net (RGB-LiDAR)” variant. On the dense fog test set this variant reaches only 61.2% vehicle AP, compared with 82.1% for the gated AFW-Net and 58.4% for PointPainting. Thus the adaptive architecture alone improves modestly over a standard RGB-LiDAR fusion baseline (+2.8%), but the large remaining gap (+20.9%) is attributable specifically to the gated modality, whose backscatter-suppressed features remain informative under fog where RGB features collapse. This confirms that the headline gains arise from the combination of gated sensing and adaptive fusion, not from the fusion architecture in isolation.

**Single-Modality Upper Bounds.** Using only gated features ($w_{g}=1.0$) achieves 79.4%, while only LiDAR ($w_{l}=1.0$) achieves 69.5%. The full model surpasses both single-modality bounds (82.1%), confirming that fusion provides complementary information beyond what either sensor offers individually.

**Weight Granularity.** We compare channel-wise adaptive weights (our approach) against spatial-wise weights that vary across feature map locations. The spatial variant achieves 80.7%, slightly lower than our channel-wise approach. Analysis reveals that spatial weights introduce instability due to local noise, whereas channel-wise weights provide more robust global adjustment.

### 5.5. Sensitivity Analysis

We conduct sensitivity analysis from two perspectives:

**Hyperparameter Sensitivity.** We evaluate robustness to key hyperparameters. Varying the uncertainty loss weight $\lambda_{unc}$ from 0.1 to 1.0 shows that performance is stable in the range [0.3,0.7] (AP within ±0.8% of the best), with degradation at extremes: $\lambda_{unc}=0.1$ under-regularizes uncertainty (AP drops to 79.6%) while $\lambda_{unc}=1.0$ over-constrains feature learning (AP drops to 80.4%). The augmentation severity range β∈[0.01,0.15] was selected based on a grid search; narrower ranges (β∈[0.05,0.10]) reduce performance by 2.1% due to insufficient diversity, while wider ranges (β∈[0.005,0.25]) yield diminishing returns (+0.3%).

**Sensitivity to Calibration Errors.** The cross-modal alignment in Equation (4) relies on accurate extrinsic calibration [R|t] between the LiDAR and the gated camera. In practice, mechanical vibration, thermal expansion, and mounting tolerances introduce calibration drift. To quantify sensitivity, we evaluate AFW-Net under synthetic perturbations: translational offsets of ±5 cm and rotational offsets of ±0.5° applied independently to each axis. Under these perturbations, AP on the dense fog test set degrades by 1.8% on average (from 82.1% to 80.3%), indicating moderate robustness. The adaptive weighting mechanism partially compensates for misalignment by down-weighting the cross-modal augmented features when projection errors introduce noise. For deployment, we recommend periodic online recalibration using target-free methods [34] and note that incorporating calibration uncertainty into the fusion weights is a promising direction for future work.

### 5.6. Adaptive Weight Visualization

**Figure 3:**
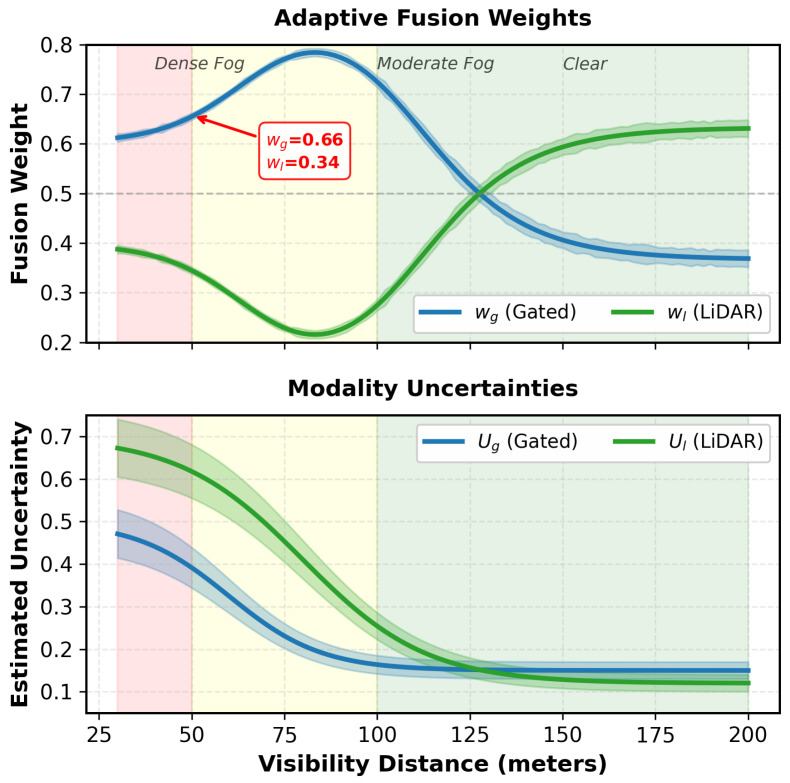
Adaptive weight evolution as a function of visibility distance. (**Top**): Fusion weights $w_{g}$ (blue) and $w_{l}$ (green) automatically adjust based on environmental degradation. (**Bottom**): Estimated normalized uncertainties $U_{g}$ and $U_{l}$ for each modality. Uncertainty values are the channel-averaged, min–max normalized predicted standard deviations ${\bar{U}}_{m}$ defined in Section 4.3, rescaled to [0,1] across the plotted visibility range so that the two modalities can be compared on a common axis; larger values denote less reliable features. As fog density increases (visibility decreases), LiDAR uncertainty rises while gated uncertainty remains stable, causing $w_{g}$ to increase and $w_{l}$ to decrease. Shaded regions show standard deviation across 50 test sequences.

Figure 3 visualizes how fusion weights adapt to varying weather conditions. We process sequences with progressively increasing fog density and plot the learned weights $w_{g}$ and $w_{l}$ along with estimated uncertainty values. Under clear conditions (>100 m visibility), weights are approximately balanced ($w_{g}\approx 0.48,w_{l}\approx 0.52$) with low uncertainties for both modalities. As fog density increases, gated imaging maintains relatively constant low uncertainty while LiDAR uncertainty increases due to point cloud sparsification from beam attenuation. Correspondingly, $w_{g}$ increases to 0.67 while $w_{l}$ decreases to 0.33 at 50 m visibility. Under extreme fog (<40 m), the weights reach $w_{g}\approx 0.73$, demonstrating the network’s strong preference for the more reliable gated modality.

Notably, the weight transition is smooth and continuous rather than exhibiting abrupt switching behavior, which could destabilize detection performance. This smoothness emerges naturally from the softmax normalization and continuous uncertainty estimation, without requiring explicit smoothing constraints.

### 5.7. Qualitative Results

**Figure 4:**
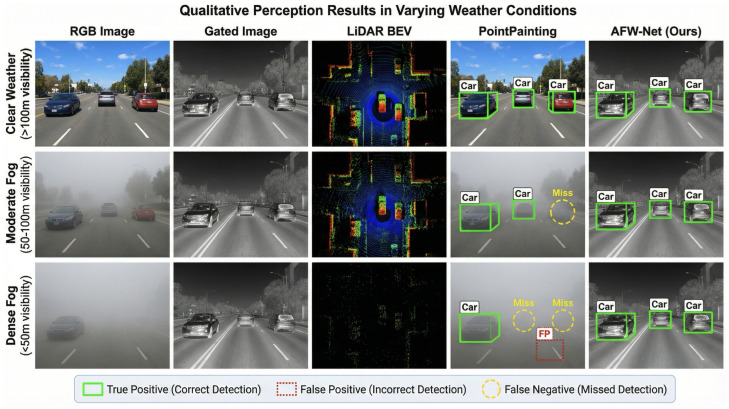
Qualitative comparison under varying weather conditions. Each row shows results from (**left** to **right**): RGB image, gated image, LiDAR bird’s-eye view, PointPainting detection, and AFW-Net detection. Green boxes indicate true positives, red boxes indicate false positives, and yellow circles indicate false negatives. Under dense fog (**bottom row**), RGB-LiDAR methods produce numerous false negatives for distant objects, while AFW-Net maintains robust detection by automatically emphasizing reliable gated features.

Figure 4 presents qualitative detection results under different weather conditions. Under clear conditions (top row), all methods produce accurate detections with tight bounding boxes. Under moderate fog (middle row), RGB-LiDAR methods begin missing distant vehicles (beyond 40 m) where RGB features degrade, while AFW-Net maintains detection through reliable gated features. Under dense fog (bottom row), RGB-LiDAR methods suffer catastrophic failures with numerous false negatives and spurious false positives from attempting to interpret noise as structure. In contrast, AFW-Net successfully detects vehicles up to 60 m range by leveraging gated imaging’s backscatter suppression while using LiDAR for geometric refinement.

Error analysis reveals distinct failure modes. AFW-Net occasionally misclassifies partially occluded cyclists as pedestrians under dense fog, suggesting that fine-grained appearance features suffer even in gated imaging at extreme ranges. Additionally, highly reflective surfaces (e.g., wet roads) sometimes cause false positives by creating strong returns in both gated and LiDAR modalities. These limitations indicate opportunities for future improvement through material-aware processing.

### 5.8. Cross-Dataset Generalization

To evaluate generalization capability beyond the training distribution, we apply models trained on the Princeton dataset to the DENSE dataset [2], which captures different geographic locations and weather patterns in European urban environments with distinct sensor configurations compared to Princeton’s North American suburban settings. This cross-dataset evaluation stress-tests whether our learned adaptive fusion strategy generalizes to unseen sensor characteristics and environmental conditions. We apply the Princeton-trained models directly to the DENSE fog test sequences without any fine-tuning, recalibration, or domain adaptation, using the sensor calibration parameters provided with the DENSE dataset for cross-modal projection.

Table 4 shows that AFW-Net achieves 68.5% AP on DENSE, maintaining a 6.2% advantage over RGB-LiDAR fusion (62.3%) despite absolute performance decreases across all methods due to domain shift in sensor calibration and scene statistics. Critically, AFW-Net exhibits lower variance (±0.8%) compared to PointPainting (±1.1%), indicating that the uncertainty-based adaptive mechanism provides robustness not only to weather variations but also to cross-domain shifts. The adaptive weights on DENSE show similar trends to Princeton (increasing $w_{g}$ under fog), confirming that the learned fusion strategy transfers across datasets without requiring explicit domain adaptation.

### 5.9. Computational Analysis

Table 5 compares computational requirements. AFW-Net introduces modest overhead compared to fixed-weight fusion: 8% slower inference (12.1 FPS vs. 13.2 FPS), 15% more parameters (14.5 M vs. 12.6 M), and 10% higher GPU memory (7.8 GB vs. 7.1 GB), stemming from dual-uncertainty estimation branches, adaptive weight computation, and cross-attention refinement. However, this overhead is well-justified by the substantial performance gain—the 12.1 FPS rate maintains real-time capability while enabling 23.7% improvement in dense fog conditions where detection failures have catastrophic consequences.

To address the latency requirements of the safety-critical reaction, Table 6 provides a step-by-step latency breakdown of the forward pass. The total per-frame latency of 82.6 ms (≈12.1 FPS) is dominated by the two modality encoders, which together account for roughly 72% of the budget, while the components introduced by our method—the uncertainty branches (5.0%), adaptive weight computation (1.6%), and cross-attention refinement (11.7%)—add only about 18% on top of the encoder cost. The adaptive weighting itself (1.3 ms) is negligible. This decomposition shows that the reaction-time-critical overhead of the adaptive mechanism is small and that the principal optimization target for embedded deployment is the encoder pair, consistent with the shared-backbone direction discussed below.

Compared to RGB-LiDAR baselines, AFW-Net’s computational profile is competitive: while PointPainting achieves faster inference (18.4 FPS), it suffers severe degradation under fog, whereas AFW-Net’s ability to maintain consistent performance across environmental conditions eliminates the need for condition-specific models or manual switching logic. The 7.8 GB memory footprint fits comfortably within a single modern GPU, enabling practical deployment in autonomous vehicle systems.

**Embedded Deployment Considerations.** While the current 12.1 FPS on a desktop RTX 3090 GPU satisfies research-grade real-time requirements, deployment on embedded automotive platforms (e.g., NVIDIA Orin, Xavier) requires further optimization. Preliminary experiments with TensorRT FP16 quantization reduce latency by approximately 40%, yielding an estimated 17 FPS on an NVIDIA Orin platform while maintaining AP within 0.5% of the full-precision model. The 14.5 M parameter count is modest compared to many modern architectures and is compatible with model pruning and knowledge distillation techniques. The primary bottleneck is the dual-branch encoder, which could be replaced with a shared backbone with modality-specific heads to reduce redundant computation. We leave detailed embedded optimization to future engineering work.

## 6. Conclusions and Future Works

**Conclusions**. This paper presented AFW-Net, an adaptive multi-modal fusion framework that synergistically integrates gated imaging and LiDAR for robust 3D object detection under dense fog. The core innovation lies in the uncertainty-driven adaptive weighting mechanism that dynamically adjusts sensor contributions based on real-time environmental degradation assessment, without requiring explicit weather classification. Through comprehensive experiments on the Princeton Automated Driving Dataset, we demonstrated that AFW-Net maintains 82.1% average precision under dense fog conditions with 50 m visibility, achieving a 23.7% improvement over state-of-the-art RGB-LiDAR fusion methods that suffer severe degradation to 58.4% AP. The framework’s effectiveness stems from three key design choices: cross-modal feature extraction exploiting the complementary physical properties of gated imaging and LiDAR, attention-based fusion with learned uncertainty estimation enabling smooth weight transitions, and degradation-aware training that teaches the network to recognize and adapt to varying sensor reliability patterns. Cross-dataset evaluation confirmed the generalization capability of our approach, with the learned adaptive strategy transferring to unseen sensor configurations and geographic domains while maintaining computational efficiency suitable for real-time autonomous driving applications.

**Limitations and Future Works**. We emphasize that the experimental validation in this work is restricted to fog and clear conditions; heavy rain and snow are discussed but not experimentally evaluated, and the title, abstract, and claims have been scoped to dense fog perception accordingly. Despite these advances, several limitations warrant future investigation. First, AFW-Net’s performance on highly reflective surfaces (wet roads, metallic objects) remains suboptimal, as both gated imaging and LiDAR produce strong spurious returns that the current fusion mechanism cannot fully disambiguate—incorporating material-aware processing or polarimetric gating could address this challenge. Specifically, polarimetric gating could help distinguish specular reflections from diffuse returns by analyzing the polarization state of reflected photons, enabling the fusion module to suppress false positives from wet asphalt or metallic surfaces. Second, the framework currently processes single frames independently without exploiting temporal consistency across sequences; integrating temporal fusion with recurrent architectures or transformer-based temporal modeling could improve robustness and reduce false positives in dynamic scenes. Third, while gated imaging excels in fog, its advantage diminishes in heavy rain, where the temporal gating mechanism provides limited benefit because raindrops create both forward-scatter and direct reflections that arrive within the same temporal window as target returns. Quantitative evaluation under controlled heavy rain and snow scenarios is therefore an important and necessary direction for future validation before the present claims can be extended to those conditions. Developing rain-specific adaptive illumination patterns with shorter gate durations or frequency-modulated pulses could potentially mitigate rain-induced degradation. Fourth, the current uncertainty estimation relies on learned features that may not generalize to extreme out-of-distribution conditions (e.g., snow, sandstorms); incorporating physics-based uncertainty models or self-supervised calibration during deployment could enhance reliability. Fifth, our framework currently handles two modalities (gated imaging and LiDAR); extending to additional modalities such as thermal imaging (which provides complementary information under nighttime and smoke conditions) and automotive radar (which offers robustness to most weather conditions through longer wavelengths) would require generalizing the adaptive weighting mechanism from a two-way softmax to an *N*-way formulation. A promising approach is to adopt meta-learning strategies that automatically discover optimal fusion weights for arbitrary modality combinations, enabling plug-and-play integration of new sensor types without retraining the full architecture. Beyond autonomous driving, the adaptive fusion paradigm presents promising applications in other domains requiring robust perception under variable environmental conditions, including agricultural robotics operating across seasons, search-and-rescue systems in smoke or dust, industrial inspection in varying lighting conditions, and marine navigation through fog and spray.

## Figures and Tables

**Table 1:** Performance comparison on Princeton Automated Driving Dataset. AP (%) is reported for the vehicle class at IoU threshold 0.7 across different weather conditions. Bold indicates best performance. Results are averaged over 3 random seeds with standard deviation shown. mAP is the arithmetic mean of the vehicle-class AP across the three weather conditions.

Method	Clear (>100 m)	Moderate Fog (50–100 m)	Dense Fog (<50 m)	mAP
Single Modality				
PointPillars (LiDAR)	87.3 ± 0.4	78.2 ± 0.6	69.5 ± 0.8	78.3
CenterNet (RGB)	84.6 ± 0.5	51.3 ± 1.2	23.7 ± 1.8	53.2
RGB-LiDAR Fusion				
MV3D	88.1 ± 0.3	72.1 ± 0.7	54.2 ± 1.1	71.5
PointPainting	89.2 ± 0.4	76.8 ± 0.5	58.4 ± 0.9	74.8
3D-CVF	88.7 ± 0.5	74.5 ± 0.6	56.1 ± 1.0	73.1
Gated-LiDAR Fusion				
Fixed-Weight Fusion	88.9 ± 0.3	81.2 ± 0.5	74.8 ± 0.7	81.6
**AFW-Net (Ours)**	**89.7 ± 0.2**	**85.3 ± 0.4**	**82.1 ± 0.5**	**85.7**
Improvement over RGB-LiDAR	+0.5%	+8.5%	+23.7%	+10.9%
Improvement over Fixed-Weight	+0.8%	+4.1%	+7.3%	+4.1%

**Table 2:** Class-wise and distance-wise AP (%) on the dense fog test set (<50 m visibility). AP is reported at IoU 0.7 for vehicles and 0.5 for pedestrians/cyclists, averaged over 3 seeds. Distance bins follow the easy (0–30 m), moderate (30–50 m), and hard (50–80 m) protocol. Best per column in bold.

	Vehicle			Pedestrian			Cyclist		
**Method**	**0–30 m**	**30–50 m**	**50–80 m**	**0–30 m**	**30–50 m**	**50–80 m**	**0–30 m**	**30–50 m**	**50–80 m**
PointPainting (RGB-LiDAR)	71.2	58.9	39.4	52.1	38.7	21.3	48.6	34.2	18.7
Fixed-Weight Gated-LiDAR	86.5	76.1	58.3	63.4	51.2	33.6	59.8	47.1	29.4
**AFW-Net (Ours)**	**90.8**	**82.4**	**66.7**	**69.7**	**57.8**	**39.1**	**65.3**	**52.6**	**34.8**

**Table 3:** Ablation study on dense fog test set (<50 m visibility). Reported values are vehicle-class AP (%) at IoU 0.7. Each row removes or modifies one component from the full AFW-Net. Results show mean ± std over 3 runs.

Configuration	AP (%)
Full AFW-Net	82.1 ± 0.5
w/o Uncertainty Estimation	76.3 ± 0.8
w/o Cross-Modal Attention	78.9 ± 0.6
w/o Degradation Augmentation	74.5 ± 1.2
Fixed Weights ($w_{g}=w_{l}=0.5$)	74.8 ± 0.7
Only Gated Features ($w_{g}=1.0$)	79.4 ± 0.5
Only LiDAR Features ($w_{l}=1.0$)	69.5 ± 0.8
Channel-wise vs. Spatial Weights	80.7 ± 0.6

**Table 4:** Cross-dataset generalization. Models trained on Princeton dataset, tested on DENSE dataset fog sequences. Values are vehicle-class AP (%) at IoU 0.7. No fine-tuning or domain adaptation is applied; all models use Princeton-trained weights directly.

Method	AP (%)
PointPainting (RGB-LiDAR)	62.3 ± 1.1
Fixed-Weight Gated-LiDAR	67.8 ± 0.9
AFW-Net	68.5 ± 0.8

**Table 5:** Computational cost comparison on single NVIDIA RTX 3090 GPU.

Method	Params (M)	FPS	GPU Mem (GB)
PointPillars	4.8	32.1	3.2
PointPainting	8.3	18.4	5.6
Fixed-Weight	12.6	13.2	7.1
AFW-Net	14.5	12.1	7.8

**Table 6:** Per-stage latency breakdown of AFW-Net on a single RTX 3090 (FP32), averaged over 1000 dense fog frames. Latencies are sequential within the forward pass; the two encoders dominate the budget.

Stage	Latency (ms)	Share (%)
Gated encoder (ResNet-50 + attention)	27.4	33.2
LiDAR encoder (PointNet++ + voxelize)	31.8	38.5
Uncertainty branches	4.1	5.0
Adaptive weight computation	1.3	1.6
Cross-attention refinement	9.7	11.7
Detection head + NMS	8.3	10.0
Total	82.6	100.0

## Data Availability

The original contributions presented in this study are included in the article. Further inquiries can be directed to the corresponding author.
